# Changes in the growth, ileal digestibility, intestinal histology, behavior, fatty acid composition of the breast muscles, and blood biochemical parameters of broiler chickens by dietary inclusion of safflower oil and vitamin C

**DOI:** 10.1186/s12917-021-02773-5

**Published:** 2021-02-04

**Authors:** Shimaa A. Amer, Wafaa A. M. Mohamed, Heba S. A. Gharib, Naif A. Al-Gabri, Ahmed Gouda, Mohamed Tharwat Elabbasy, Ghada I. Abd El-Rahman, Anaam E. Omar

**Affiliations:** 1 Department of Nutrition & Clinical Nutrition, Faculty of Veterinary Medicine, Zagazig University, Zagazig, 44511 Egypt; 2 Department of Clinical Pathology, Faculty of Veterinary Medicine, Zagazig University, Zagazig, 44511 Egypt; 3 Department of Veterinary Public Health, Faculty of Veterinary Medicine, Zagazig University, Zagazig, 44511 Egypt; 4 Pathology Department, Faculty of Veterinary Medicine, Thamar University, Dahamar, Yemen; 5 Department of Animal Production, National Research Centre, Dokki, 12622 Egypt; 6 College of Public Health and Molecular Diagnostics and Personalized Therapeutics Center (CMDPT) Hail University, Hail, 2440 Saudi Arabia; 7 Food Control Department, Faculty of Veterinary Medicine, Zagazig University, Zagazig, 44519 Egypt

**Keywords:** Broiler chicken, Safflower oil, Vitamin C, Ileal digestibility, Fatty acid composition

## Abstract

**Background:**

The effects of safflower oil and vitamin C (Vit. C) inclusion in broiler chicken diets on the growth performance, apparent ileal digestibility coefficient “AID%” of amino acids, intestinal histology, behavior, carcass traits, fatty acid composition of the breast muscle, antioxidant and immune status for a 35-day feeding period were evaluated. A total of 300 three-day-old Ross chicks (58.25 g ± 0.19) were randomly allotted in a 2 × 3 factorial design consisting of two levels of vitamin C (0 and 400 mg/kg diet) and three levels of safflower oil (0, 5, and 10 g/kg diet).

**Results:**

An increase in the final body weight, total body weight gain, total feed intake, and the relative growth rate (*P* <  0.05) were reported by safflower oil and vitamin C inclusion. Dietary supplementation of safflower oil and vitamin C had a positive effect (*P <  0.05*) on the ingestive, resting, and feather preening behavior. Vitamin C supplementation increased (*P <  0.05*) the AID% of lysine, threonine, tryptophan, arginine, and valine. Safflower inclusion (10 g/kg) increased (*P <  0.05*) the AID% of methionine and isoleucine. Safflower oil inclusion increased (*P <  0.05*) the levels of stearic acid, linoleic acid, saturated fatty acids, and omega-3 fatty acids (ω-3) in the breast muscle. In contrast, the supplementation of only 10 g of safflower oil/kg diet increased (*P = 0.01*) the omega-3/omega-6 (ω-3/ω-6) fatty acids ratio. Vit. C supplementation increased (*P <  0.05*) the CAT serum levels, SOD, and GSH enzymes. Dietary supplementation of safflower oil and vitamin C improved the intestinal histology. They increased the villous height and width, crypt depth, villous height/crypt depth ratio, mucosal thickness, goblet cell count, and intra-epithelium lymphocytic lick cell infiltrations. The serum levels of IgA and complement C3 were increased (*P <  0.01*) by Vit. C supplementation and prominent in the 400 vit. C +  10 safflower Oil group.

**Conclusion:**

A dietary combination of safflower oil and vitamin C resulted in improved growth rate, amino acids AID%, intestinal histology, welfare, immune and antioxidant status of birds, and obtaining ω-3 and linoleic acid-enriched breast muscles. The best inclusion level was 400 vit. C +  10 safflower Oil.

## Background

The fatty acids “FA” composition of broiler carcass can be modified by enriching it with high levels of essential polyunsaturated fatty acids (PUFA) through the dietary supplementation of plant seed oils [1–4]. Plant-rich oils contain high levels of polyunsaturated fatty acids (PUFA) but vary in the composition of essential FA. Consequently, these oils improve the PUFA carcass content with a different effect on the composition of overall fatty acid carcasses when fed to chicken [3, 5].

The reported benefits of polyunsaturated fatty acids on human health have increased the interest in foods containing these fatty acids in higher concentrations [3, 6–8]. The body’s FA composition is mainly dependent on the contribution of hepatic lipid and external dietary fat as a source of FA deposits in the body. Dietary supplements affect each of these parameters and work either in an identical or reverse manner, depending on the diet composition. Fatty acids C18:2 and C18:3 are indispensable and cannot be synthesized; they are ingested through the dietary fat. The biochemical pathway of FA biosynthesis is affected by the dietary FA availability.

Safflower has been cultured locally for its oil, meal, and flower [9]. The normal-hull seeds contain 5–8% moisture, 27–32% oil, 14–15% protein, 32–40% crude fiber, and 2–7% ash [10, 11]. Safflower is one of the preeminent crops with variations in fatty acid composition in seed oil [12]. Common safflower oil encloses around 71–75% linoleic acid, 2–3% stearic acid, 16–20% oleic acid, and 6–8% palmitic acid [13]. Safflower oil contains 79% of fatty acids as C18:2 (n-6), 11% as C18:1 (n-9), and 0.5% as C18:3 (n-3) [14]. Conjugated linoleic acid (CLA) is a broad name for the positional and arithmetical isomers of linoleic acid (9 cis, 12 cis octadecadienoic acid; 18:2 (n-6)). In various mammalian species and chickens, it is observed that the fat deposition decreases by dietary conjugated linoleic acid [15, 16] that enhances skeletal muscle fatty acid β-oxidation [17, 18]. This effect is demonstrated by the up-regulation of the carnitine palmitoyltransferase I (CPT-I, the main enzyme for β-oxidation) in skeletal muscles [19–21].

The main reason for meat and meat product deterioration is fat oxidation. High levels of PUFA in the poultry meat make it more liable to fatty acid oxidation; this oxidation can be increased by particular feeding strategies [22]. Although dietary handling results in increasing muscle tissue level, unsaturation increases the poultry meat vulnerability to oxidative degradation [23]. This leads to the formation of many products, including short-chain aldehydes, ketones, and other oxygenated compounds that can badly influence the overall meat quality and decrease its nutritional value [24]. Therefore, food supplements are an easy and suitable approach to include antioxidant compounds in chicken meat.

Vitamin C acts as an antioxidant because it protects the cellular components from free radical damage. Like most monogastric animals, Poultry synthesizes sufficient ascorbic acid for normal development and growth [25, 26]. Attia, et al. [27] demonstrated a positive effect of vitamin C supplementation, both in isolation and in combination with vitamin E and probiotics, in alleviating the adverse effects of chronic heat stress on the growth and immunity of broiler chickens. Moreover, Vitamin C supplementation is reported to effectively reduce the serum cholesterol concentration and the expression of heat shock protein 70 (HSP70) gene in the heart and liver of heat-stressed broilers [28]. Ascorbic acid reduces the formation of tocopherol radicals during free radical scavenging in the metabolism. The rejuvenated vitamin E molecule works as an antioxidant again or it is stored [29]. Thus, dietary vitamin C improves the antioxidant status of chicken tissues [30, 31]. Vitamin C plays a significant role in interacting with all types of aggressive oxygen molecules under essentially inactive radical formation and transferring the radical equivalent from the fatty stages to the water molecules [32, 33].

To our knowledge, no information is available on the effect of including a combination of safflower oil and vitamin C in broiler chicken diets. Therefore, this study aims to assess the impact of safflower oil and vitamin C inclusion in broiler chicken diets on the growth performance, amino acid ileal digestibility, carcass traits, behavior, breast muscle FA composition, intestinal histology, and immunological and antioxidant status of broiler chickens.

## Results

### Growth performance

Table 1 shows the effect of dietary inclusion of safflower oil and vitamin C on broiler chickens’ growth performance. In the starter stage, dietary inclusion of 400 mg vitamin C/kg diet (Vit.C400) resulted in a significantly higher BW (*P = 0.02*), BWG (*P = 0.02*), and FI (*P = 0.04*). The safflower oil level had no significant effect on the BW, BWG, FI, and FCR (*P > 0.05*). The interaction between vitamin C and safflower oil resulted in a significant increase in the BW, BWG, and FI (*P <  0.05*) in the groups fed diets supplemented with vitamin C and safflower oil.

**Table 1:** The effect of inclusion of safflower oil and vitamin C in the broiler chicken diet on the growth performance

Item	Vitamin C level (mg/kg diet)		Safflower oil level (gm/kg diet)			Vitamin C × Safflower oil						SEM	*P-value*		
	0	400	0	5	10	0Vit.C+ 0Saff. Oil	0Vit.C + 5 Saff. Oil	0Vit.C+ 10 Saff. Oil	400Vit.C+ 0Saff. Oil	400Vit.C+ 5 Saff. Oil	400Vit.C+ 10Saff. Oil		Vit. C	Saff. oil	Vit. C x Saff. oil
Int.wt(g)	57.44	58.24	57.45	58.33	58.74	57.56	58.33	58.88	57.78	58.33	58.61	0.19	0.96	0 .07	0.42
Starter period															
BW (g)	203.06^b^	222.31^a^	201.67	219.86	216.52	181.13^b^	219.16^a^	208.89^a^	222.22^a^	220.55^a^	224.16^a^	4.38	0.02	0.20	< 0.01
BWG(g)	145.61^b^	164.07^a^	144.22	161.52	157.77	122.91^b^	160.83^a^	150^a^	164.44^a^	162.22^a^	165.55^a^	4.35	0.02	0.21	0.008
FI(g)	212.81^b^	230.20^a^	207.67	228.47	228.38	188.23^b^	227.66^a^	222.55^a^	227.11^a^	229.28^a^	234.22^a^	4.42	.045	0.07	0.007
FCR	1.46	1.40	1.43	1.42	1.45	1.52	1.41	1.48	1.38	1.42	1.41	0.01	.079	0.75	0.29
Grower period															
BW(g)	775.92	818.14	757.22^b^	820.41^a^	813.47^a^	718.89^b^	807.50^a^	801.39^a^	795.55^a^	833.33^a^	825.55^a^	11.36	0.06	0.03	0.02
BWG(g)	572.86	595.83	555.54^b^	600.55^a^	596.94^a^	537.75^b^	588.33^a^	592.5^a^	573.33^ab^	612.77^a^	601.39^ab^	7.87	0.15	0.02	0.06
FI(g)	945.38	962.42	870.41^b^	976.83^a^	1014.45^a^	835.33^c^	965.35^ab^	1035.45^a^	905.49^bc^	988.31^ab^	993.44^ab^	19.022	0.66	< 0.01	< 0.01
FCR	1.64	1.61	1.56	1.62	1.70	1.55	1.64	1.74	1.58	1.61	1.66	0.02	0.61	0.11	0.42
Finisher period															
BW(g)	1793.38	1900.68	1717.07^b^	1903.96^a^	1920.06^a^	1586.22^b^	1904.66^a^	1889.26^a^	1847.93^a^	1903.26^a^	1950.86^a^	31.06	0.08	< 0.01	0.00
BWG(g)	1017.45	1082.54	959.85^b^	1083.54^a^	1106.59^a^	867.33^b^	1097.16^a^	1087.87^a^	1052.37^a^	1069.93^a^	1125.31^a^	21.70	0.13	< 0.01	0.00
FI(g)	1799.01	1877.50	1747.26	1885.55	1881.94	1691.74	1863.88	1841.38	1802.78	1907.22	1922.5	30.65	0.21	0.10	0.29
FCR	1.78	1.73	1.83	1.74	1.70	1.95	1.69	1.69	1.71	1.78	1.70	0.03	0.50	0.25	0.14
Overall performance															
FBW(g)	1793.38	1900.68	1717.07^b^	1903.96^a^	1920.06^a^	1586.22^b^	1904.66^a^	1889.26^a^	1847.93^a^	1903.26^a^	1950.86^a^	31.06	0.08	< 0.01	< 0.01
BWG(g)	1735.12	1842.44	1659.40^b^	1861.31^a^	1845.63^a^	1528.66^b^	1846.33^a^	1830.38^a^	1790.15^a^	1844.93^a^	1892.25^a^	30.96	0.08	< 0.01	< 0.01
FI(g)	2957.20	3070.12	2825.34^b^	3090.86^a^	3124.78^a^	2715.31^b^	3056.90^a^	3099.39^a^	2935.38^a^	3124.81^a^	3150.17^a^	49.30	0.26	0.01	< 0.01
FCR	1.70	1.66	1.70	1.67	1.68	1.77	1.65	1.69	1.63	1.69	1.66	0.02	0.32	0.83	0.53
RGR	187.33^b^	188.09^a^	186.93^b^	188.11^a^	188.10^a^	185.99^b^	188.11^a^	187.90^a^	187.87^a^	188.10^a^	188.30^a^	0.19	0.05	0.01	< 0.01

^a, b, c^ Means within the same row carrying different superscripts are significantly different (*P* <  0.05). *BW* body weight, *BWG* body weight gain, *FI* feed intake, *FCR* feed conversion ratio, *PER* protein efficiency ratio, *RGR* relative growth rate

In the grower stage, the BW, BWG, and FI increased significantly by safflower oil inclusion (*P <  0.05*). The dietary inclusion of vitamin C had no significant effect on the BW, BWG, FI, and FCR (*P > 0.05*). The bird weights increased significantly (*P = 0.02*) by feeding them a diet containing vitamin C and safflower oil. The BWG and FI of birds fed on a diet containing safflower oil alone or a combination of safflower oil and vitamin C were significantly higher (*P <  0.05*).

In the finisher stage, dietary safflower oil inclusion resulted in a significant increase in the birds’ BW and BWG (*P <  0.01*). The dietary inclusion of vitamin C produced no significant effect on the BW, BWG, FI, and FCR (*P > 0.05*). Interaction between vitamin C and safflower oil resulted in a significant increase in the BW and BWG (*P <  0.05*) in the groups of birds fed diets supplemented with vitamin C and safflower oil. The final BW, total BWG, and total FI increased significantly (*P <  0.05*) upon the inclusion of safflower oil and vitamin C. The relative growth rate “RGR” increased significantly (*P <  0.01*) by the dietary inclusion of safflower oil and vitamin C. No significant difference was observed in the FCR by the inclusion of safflower oil and vitamin C throughout the experimental periods (*P > 0.05*).

### Apparent ileal digestibility coefficient “AID%” of amino acids

Table 2 highlights the effect of including safflower oil and vitamin C in broiler chicken diets on the AID% of various amino acids. Dietary supplementation with a 400 mg/kg diet of vitamin C significantly increased (*P <  0.05*) the AID% of lysine, threonine, tryptophan, arginine, and valine compared to the zero inclusion level (a diet without any supplementation). However, it did not have any significant effect (*P > 0.05*) on the AID% of methionine, leucine, and isoleucine. Dietary inclusion of a 10 g/kg diet of safflower significantly increased the AID% of methionine and isoleucine (*P <  0.05*) and decreased the AID% of leucine (*P <  0.01*) compared to zero inclusion level. The interaction between vitamin C and safflower oil resulted in a significant increase in (a) the lysine AID% (*P <  0.01*) in the 0Vit.C + 5 Saff. Oil, 400Vit.C + 0 Saff. Oil, 400Vit.C + 5 Saff. Oil, and 400Vit.C + 10 Saff. Oil groups; (b) the methionine AID% (*P <  0.01*) in the 0Vit.C + 5 Saff. Oil, 0Vit.C + 10 Saff. Oil, 400Vit.C + 0 Saff. Oil, and 400Vit.C + 10 Saff. Oil groups; (c) the threonine AID% (*P <  0.01*) in the 0Vit.C + 5 Saff. Oil, 0Vit.C + 10 Saff. Oil, 400Vit.C + 0 Saff. Oil, and the 400Vit.C + 10 Saff. Oil groups; (d) the tryptophan AID% (*P <  0.01*) in the 400Vit.C + 10 Saff. Oil group; (e) the arginine AID% (*P <  0.01*) in the 400Vit.C + 0 Saff. Oil group; (f) the leucine AID% (*P <  0.01*) in the 400Vit.C + 0 Saff. Oil group; and (g) the isoleucine AID% (*P <  0.01*) in the 0Vit.C + 10 Saff. Oil, 400Vit.C + 5 Saff. Oil, and the 400Vit.C + 10 Saff. Oil groups; and (g) the valine AID% (*P* = 0.00) in the 400Vit.C + 5 Saff. oil and 400Vit.C + 10 Saff. Oil groups. The interaction resulted in a significant decrease in the 0Vit.C + 5 Saff. Oil group.

**Table 2:** The effect of inclusion of safflower oil and vitamin C in the broiler chicken diet on the apparent ileal digestibility coefficient (AID%) of amino acids

Item	Vitamin C level (mg/kg diet)		Safflower oil level (gm/kg)			Vitamin C × Safflower oil						SEM	*P-value*		
	0	400	0	5	10	0Vit.C+ 0 Saff. Oil	0Vit.C + 5 Saff. Oil	0Vit.C+ 10 Saff. Oil	400Vit.C+ 0 Saff. Oil	400Vit.C+ 5 Saff. Oil	400Vit.C+ 10 Saff. Oil		Vit. C	Saff. oil	Vit.C x Saff. oil
Lysine	88.54^b^	88.91^a^	88.78	88.78	88.61	88.40^d^	88.66^c^	88.57^cd^	89.16^a^	88.91^b^	88.66^c^	0.16	< 0.01	0.46	< 0.01
Methionine	84.51	84.10	83.96^b^	84.06^ab^	84.90^a^	83.54^c^	84.79^b^	85.21^a^	84.38^b^	83.33^c^	84.58^b^	0.06	0.22	0.03	< 0.01
Threonine	84.69^b^	85.27^a^	85.12	84.94	84.88	85.19^ab^	84.57^c^	84.32^c^	85.06^b^	85.31^ab^	85.43^a^	0.10	< 0.01	0.60	< 0.01
Tryptophan	86.54^b^	87.99^a^	87.27	87.03	87.51	87.03^bcd^	86.06^d^	86.54^cd^	87.51^abc^	87.99^ab^	88.47^a^	0.22	< 0.01	0.70	< 0.01
Arginine	89.05^b^	89.31^a^	89.29	89.08	89.16	89.08^bc^	88.99^c^	89.08^bc^	89.51^a^	89.16^bc^	89.25^b^	0.04	< 0.01	0.13	< 0.01
Valine	84.61^b^	85.13^a^	84.83	84.78	85.00	84.83^b^	84.39^c^	84.61^bc^	84.83^b^	85.16^a^	85.38^a^	0.06	< 0.01	0.55	< 0.01
Leucine	90.07	90.16	90.21^a^	90.13^ab^	90.01^b^	90.05^b^	90.11^b^	90.00^b^	90.37^a^	90.11^b^	90.02^b^	0.03	0.15	< 0.01	< 0.01
Isoleucine	85.07	85.23	84.91^b^	85.09^b^	85.45^a^	84.91^b^	84.91^b^	85.39^a^	84.91^b^	85.27^a^	85.51^a^	0.08	0.22	< 0.01	< 0.01

^a, b, c^ Means within the same row carrying different superscripts are significantly different at (*P* <  0.05)

### Histological findings

**Fig. 1:**
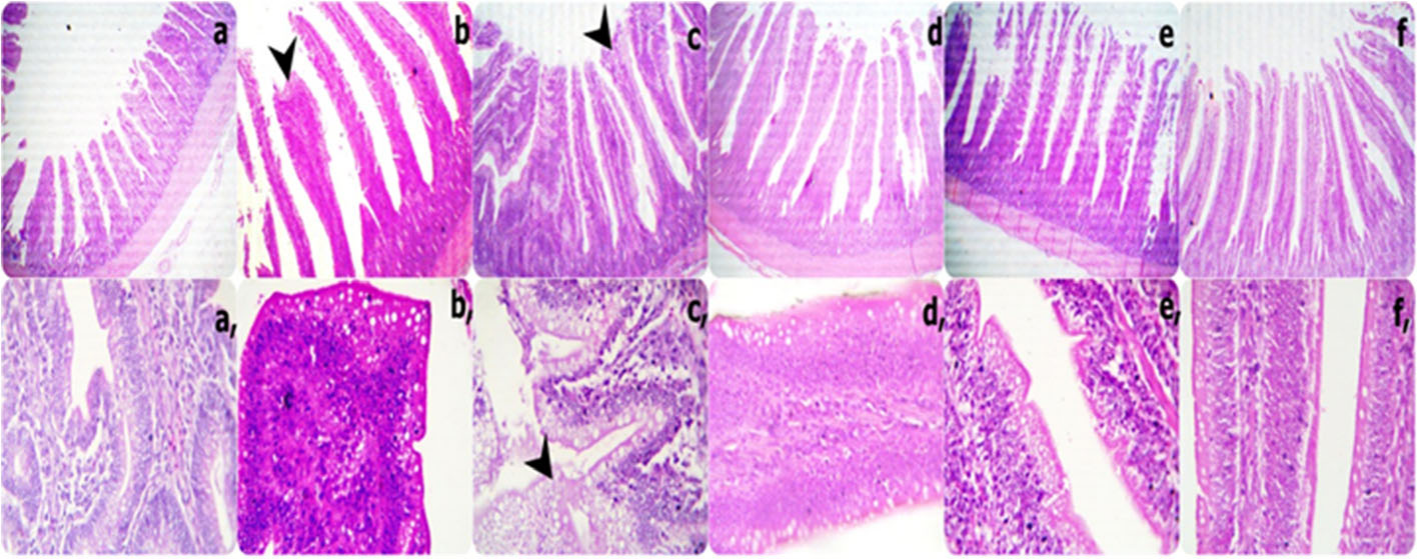
Representative photomicrograph of the low (40X) and high (400X) magnification H&E stained duodenums segment sections showing short and thick villi with limitation goblet cell metaplasia besides increasing inflammatory cells in the lamina propria in 0Vit.C+ 0Saff. Oil group; tall and thin villi with a few broad tips in a few villi without goblet cell metaplasia in 0Vit.C + 5 Saff. Oil group; marked brad tips and serrated surfaces with marked goblet cell metaplasia besides mild adhesions in 0Vit.C+ 10Saff. Oil group; more healthy which characterized by gradual increase tall and thin villi without goblet cells metaplasia in each 400Vit.C + 0Saff. Oil, 400Vit.C + 5Saff. Oil, and 400Vit.C + 10Saff. Oil groups respectively except increase intra-epithelium lymphocytic lick cells infiltrations in 400Vit.C+ 10Saff. Oil group. (a and a: 0Vit.C+ 0Saff. Oil group, b, and b: 0Vit.C + 5 Saff. Oil group, c, and c:0Vit.C+ 10Saff. Oil group: d and d: 400Vit.C + 0Saff. Oil, e and e: 400Vit.C + 5Saff. Oil, f and f: 400Vit.C+ 10Saff. Oil group)

Sections from the duodenal segments displayed (i) short and thick villi with limited goblet cell metaplasia besides increased inflammatory cells in the lamina propria in the 0Vit.C+ 0Saff. Oil group; (ii) tall and thin villi with few broad tips in certain villi without goblet cell metaplasia in the 0Vit.C + 5 Saff. Oil group; (iii) marked broad tips and serrated surfaces with marked goblet cell metaplasia besides mild adhesions in the 0Vit.C+ 10Saff. Oil group. The duodenal sections from the 400Vit.C + 0Saff. Oil, 400Vit.C + 5Saff. Oil, and 400Vit.C + 10Saff. Oil groups appeared healthier and were characterized by a gradual increase in tall and thin villi without goblet cell metaplasia. The exception was an increased infiltration of the intra-epithelial lymphocytic lick cells in the 400Vit.C+ 10Saff. Oil group **(**Fig. 1**).**

**Fig. 2:**
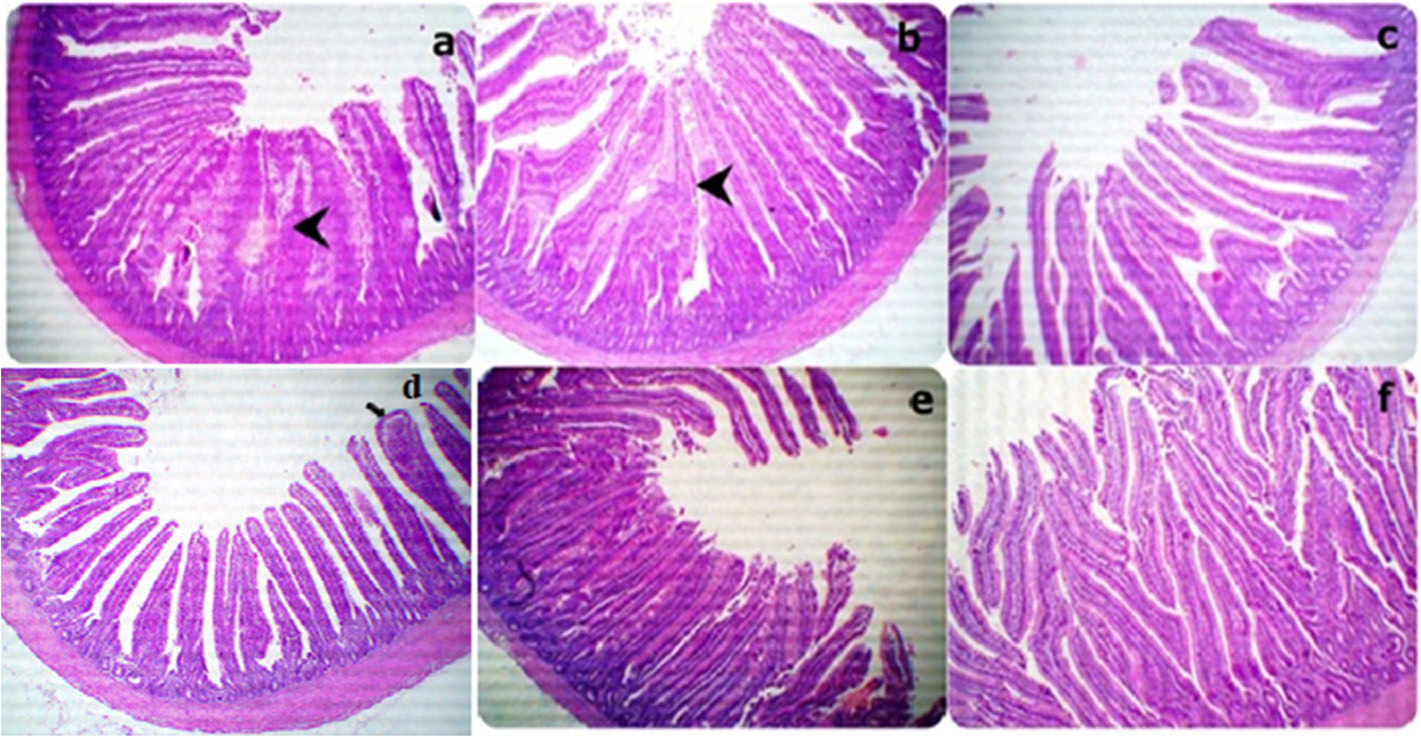
Representative photomicrograph of the (40X) magnification H&E stained jejunum segment sections showing a little denuded villi parts in the lumen beside marked adhesions of the villi in both a and b groups: gradually increased of the tall villi in each c, d, e and f groups respectively. (**a**: 0Vit.C+ 0Saff. Oil group, **b**: 0Vit.C + 5 Saff. Oil group, **c**: 0Vit.C+ 10Saff. Oil group: **d**: 400Vit.C + 0Saff. Oil, **e**: 400Vit.C + 5Saff. Oil, **f**: 400Vit.C+ 10Saff. Oil group)

Sections from the jejunal segments displayed some denuded villi parts in the lumen besides marked adhesions of the villi in both 0Vit.C+ 0Saff. Oil and 0Vit.C + 5 Saff. Oil groups. A gradual increase was observed in the tall villi in each of the 0Vit.C+ 10Saff. Oil, 400Vit.C + 0Saff. Oil, 400Vit.C + 5Saff. Oil, and the 400Vit.C+ 10Saff. Oil groups, respectively **(**Fig. 2**).**

**Fig. 3:**
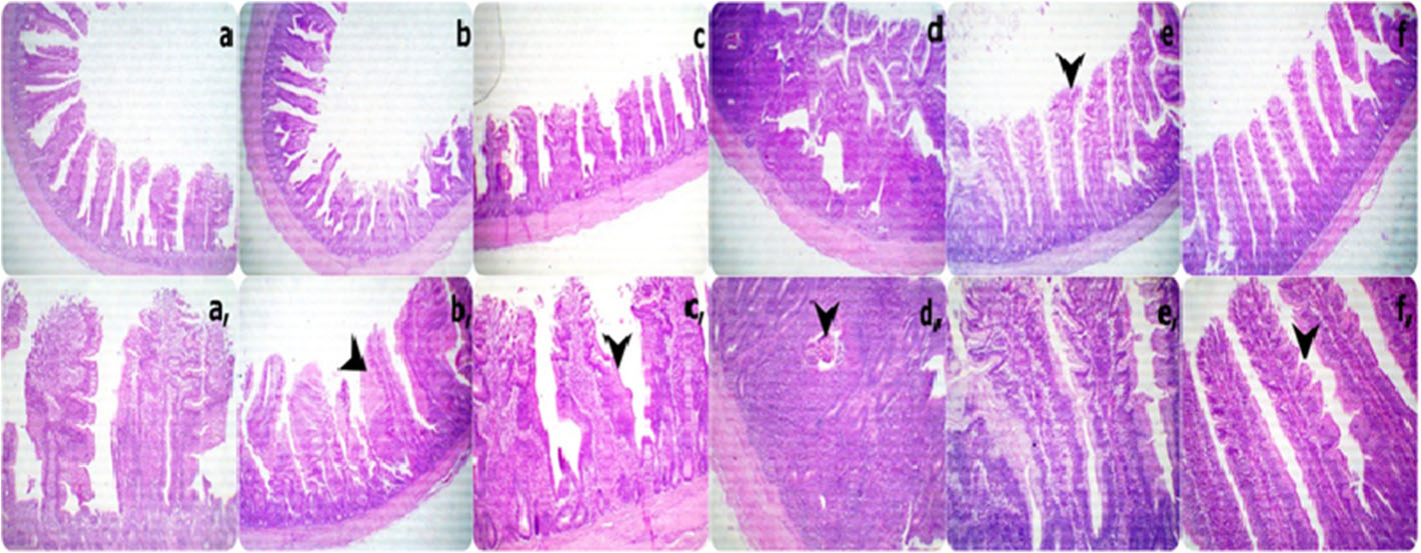
Representative photomicrograph of the low (40X) and high (100X) magnification H&E stained ileum segment sections showing short and thick villi with increase goblet cells metaplasia with hypertrophic enterocytes **a**, **b** and **c** groups: severe active associated lymphocytic aggregations (Pyre’s Patches) with necrotic parts (arrowhead) in d group: tall and thin villi with increase absorptive surfaces which represented by brad tips and serrated surfaces due to increase proliferated enterocytes (arrowhead) in both e and f groups. (a and a: 0Vit.C+ 0Saff. Oil group, b, and b: 0Vit.C + 5 Saff. Oil group, c, and c: 0Vit.C+ 10Saff. Oil group: d and d: 400Vit.C + 0Saff. Oil, e and e: 400Vit.C + 5Saff. Oil, f and f: 400Vit.C+ 10Saff. Oil group)

Sections from the ileal segments displayed (i) short and thick villi with increased goblet cell metaplasia and hypertrophic enterocytes in the 0Vit.C+ 0Saff. Oil, 0Vit.C + 5Saff. Oil, and 0Vit.C + 10Saff. Oil groups; (ii) severe acute associated lymphocytic aggregations (Peyer’s Patches) with necrotic parts in the 400Vit.C + 0Saff. Oil group; and (iii) tall and thin villi with increased absorptive surfaces represented by broad tips and serrated surfaces due to increased proliferating enterocytes in both the 400Vit.C + 5Saff. Oil and the 400Vit.C+ 10Saff. Oil groups **(**Fig. 3**).**

### Morphometric measures of the small intestine

The effect of vitamin C on the morphometric measures of the intestine is shown in Table 3. Dietary supplementation with 400 mg/kg diet of vitamin C resulted in a significant increase (*P <  0.05*) in the duodenal and jejunal mucosal depth and ileal crypt depth. Moreover, it caused a significant decrease (*P <  0.05*) in the duodenal and jejunal goblet cell count and intra-epithelium lymphocytic lick cells infiltration and decreased the jejunal and ileal villous height/crypt depth ratio significantly (*P* <  0.05) when compared to the measurements in the non-supplemented diet.

**Table 3:** The effect of the inclusion of safflower oil and vitamin C in the broiler chicken diet on the intestine’s morphometric measures

Item	Vitamin C level (mg/kg diet)		Safflower oil level (gm/kg diet)			Vitamin C × Safflower oil						SEM	*P*-value		
	0	400	0	5	10	0Vit.C+ 0Saff. Oil	0Vit.C + 5 Saff. Oil	0Vit.C+ 10 Saff. Oil	400Vit.C+ 0Saff. Oil	400Vit.C+ 5 Saff. Oil	400Vit.C+ 10Saff. Oil		Vit. C	Saff. oil	Vit. C x Saff. oil
Duodenum															
Villous height	1062.06	1153.6	777.17^b^	1314.63^a^	1231.77^a^	749.15^b^	1461.17^a^	975.87^b^	889.01^b^	1149.3^ab^	1487.6^a^	80.75	0.52	< 0.01	< 0.01
Villous width	176.40	134.42	85.84	181.84	198.56	15.09^b^	260.98a	253.14^a^	140.30^ab^	111.89^ab^	143.97^ab^	26.19	0.36	0.08	< 0.01
Crypt depth	286.02	241.77	98.61^b^	482.31^a^	210.76^b^	33.64^d^	616.55^a^	207.86^c^	189.42^cd^	366.49^b^	213.66^bc^	45.77	0.58	0.00	< 0.01
VH/CD	10.12	5.09	13.90^a^	2.94^b^	5.97^b^	22.91^a^	2.44^c^	5.01^ab^	4.89^ab^	3.44^ab^	6.93^b^	1.72	0.09	< 0.01	< 0.01
Mucosal depth	152.98^b^	182.71^a^	92.55^b^	236.44^a^	174.56^ab^	21.52^e^	301.97^a^	135.46^d^	163.34^cd^	173.75^c^	213.66^b^	20.48	0.41	< 0.01	< 0.01
GCC	121.42^a^	15.33^b^	20.13b	25.13^b^	159.88^a^	11.75^b^	140.75^b^	311.75^a^	25.60^b^	8.00^b^	8.00^b^	27.22	0.02	0.01	< 0.01
IELI	46.75^a^	22.00^b^	15.25	49.25	38.63	9.00^b^	77.50^a^	53.75^ab^	19.20^b^	24.67^b^	23.50^ab^	7.59	0.05	0.09	< 0.01
Jejunum															
Villous height	880.92	865.20	895.05	843.87	880.27	840.36	797.62	1004.79	937.34	890.90	755.76	38.96	0.82	0.82	0.28
Villous width	167.13	128.81	57.75^b^	214.22^a^	171.94^a^	10.16^c^	282.80^a^	208.44^ab^	103.01^bc^	162.95^b^	135.45^b^	22.71	0.34	< 0.01	< 0.01
Crypt depth	198.27	214.03	117.52^b^	259.83^a^	241.09^a^	23.71^b^	311.45^a^	259.65^a^	197.82^a^	246.38^a^	222.53^a^	26.00	0.73	0.01	< 0.01
VH/CD	14.98^a^	4.29^b^	20.95^a^	3.81^b^	4.15^b^	37.46^a^	2.58^b^	4.89^b^	4.43^b^	5.05^b^	3.40^b^	3.13	0.04	< 0.01	< 0.01
Mucosal depth	163.05^b^	189.99^a^	115.49	207.62	206.45	19.63^d^	279.15^a^	190.37^bc^	187.82^ab^	150.21^c^	202.53^ab^	20.89	0.46	0.05	< 0.01
GCC	220.25^a^	11.08^b^	107.38	115.75	123.88	204.50^a^	221.25^a^	235.00^a^	9.00^b^	12.33^b^	12.75^b^	29.32	0.00	0.96	< 0.01
IELI	82.75^a^	22.33^b^	23.75	65.63	68.25	17.00^b^	109.00^a^	122.25^a^	32.40^b^	16.33^b^	14.25^b^	11.40	0.001	0.11	< 0.01
Ileum															
Villous height	629.71	691.81	500.30^c^	651.94^b^	830.04^a^	436.86^c^	625.73^abc^	826.55^a^	578.01^bc^	692.5^ab^	833.52^a^	39.06	0.37	0.00	< 0.01
Villous width	187.66	176.16	111.90b	207.60ab	226.22a	20.44c	226.46^ab^	316.08^a^	188.99^b^	207.83^b^	136.37^b^	23.50	0.78	0.04	< 0.01
Crypt depth	110.09^b^	179.74^a^	94.61^b^	166.83^a^	173.30^a^	33.82^c^	127.34^b^	169.10^b^	175.32^ab^	190.10^a^	177.5^ab^	14.40	< 0.01	0.01	< 0.01
VH/CD	7.68^a^	4.02^b^	8.38^a^	4.32^b^	4.84^b^	12.95^a^	5.19^b^	4.89^b^	3.82^b^	3.44^b^	4.80^b^	0.86	0.01	0.04	< 0.01
Mucosal depth	152.91	159.03	94.55^b^	179.00^a^	194.37^a^	33.69^b^	213.80^a^	211.25^a^	149.55^a^	150.21^a^	177.50^a^	16.89	0.84	< 0.01	< 0.01
GCC	219.33	158.92	127.00^b^	336.50^a^	103.88^b^	245.50^ab^	375.25^a^	37.25^bc^	87.00^c^	263.33^a^	170.5^abc^	38.18	0.37	< 0.01	0.01
IELI	79.58	253.58	363.25	93.63	42.88	7.25^b^	160.00^b^	71.50^b^	578.20^a^	31.67^b^	14.25^b^	50.15	0.16	0.20	0.01

^a, b, c^ Means within the same row carrying different superscripts are significantly different (*P <  0.05*). *VH/CD* villous height/crypt depth ratio, *GCC* Goblet cells count, *IELI* Intra-epithelium lymphocytic lick cell infiltrations

The duodenal villous height was significantly increased (*P <  0.05*) in the 5 and 10saff. Oil groups compared to that in the 0saff. Oil group. Dietary supplementation with 5saff. Oil resulted in a significant increase (*P <  0.05*) in the duodenal crypt depth and mucosal thickness compared to that in the 0saff.oil group. There was a significant increase (*P <  0.05*) in the duodenal goblet cell count in the 10saff.oil group compared to that in the 0saff. Oil group. A significant increase (*P <  0.05*) in the jejunal villous width and crypt depth was found in the 5 and 10saff. Oil groups compared to that observed in the 0saff. Oil group. Dietary supplementation with 5 and 10saff. Oil resulted in a significant increase (*P <  0.05*) in the ileal villous height, crypt depth, and mucosal thickness compared to that in the 0saff.oil group. A significant increase in the ilea villous width (*P <  0.05*) was found in 10saff. Oil group, and a significant increase in the ilea goblet cell count (*P <  0.05*) was found in 5saff.oil group compared to the 0saff.oil group. Safflower supplementation at both levels decreased the villous height/crypt depth ratio significantly (*P <  0.05*) in all small intestine parts compared to that observed in the non-supplemented diet.

The interaction between vitamin C and safflower oil resulted in a significant increase (*P <  0.01*) in (i) the duodenal villous height in the 0Vit.C + 5saff. Oil and 400Vit.C + 10saff. Oil groups; (ii) the duodenal villous width in the 0Vit.C + 5saff. Oil and 0Vit.C + 10saff. Oil groups; and (iii) the duodenal crypt depth and mucosal thickness in all the experimental groups compared to the 0Vit.C + 0saff. oil group. However, the highest crypt depth and mucosal thickness were found in the 0Vit.C + 5saff. Oil group. Moreover, the interaction between vitamin C and safflower oil resulted in a significant increase (*P <  0.01*) in (iv) the duodenal goblet cell count in the 0Vit.C + 10saff. Oil group and (v) the duodenal intra-epithelium lymphocytic lick cells infiltrations in the 0Vit.C + 5saff. Oil group compared to the 0Vit.C + 0saff. Oil group; (vi) the jejunal villus width, crypt depth, and mucosal thickness in all diets that included vitamin C and safflower oil compared to the 0Vit.C + 0saff. Oil group; (vii) the jejunal intra-epithelium lymphocytic lick cells infiltration in the 0Vit.C + 5saff. Oil and 0Vit.C + 10saff. Oil groups compared to the 0Vit.C + 0saff. Oil group; (viii); the ileal villous height in the 0Vit.C + 10saff. Oil and 400Vit.C + 10saff. Oil groups; (ix) the ileal villus width, crypt depth, and mucosal thickness in all experimental groups compared to the 0Vit.C + 0saff. Oil group; and (x) the ileal intra-epithelium lymphocytic lick cells infiltration in the 400Vit.C + 0saff. Oil group compared to the 0Vit.C + 0saff. Oil group. However, a significant decrease (*P <  0.01*) in the jejunal goblet cell count was observed in the 400Vit.C + 0saff.Oil, 400Vit.C + 5saff.Oil, and 400Vit.C + 10saff.oil groups. Also, a significant decrease (*P = 0.01*) was observed in the ileal goblet cell count in the 400Vit.C + 0saff. Oil group; The villous height/crypt depth ratio (*P <  0.05*) was decreased in the duodenum in the 0Vit.C + 5saff.Oil and 400Vit.C + 10saff. Oil groups and in the jejunum and ileum in all experimental groups compared with the 0Vit.C + 0saff.Oil group.

### Carcass traits

The carcass traits represented as “% relative to the carcass weight” are shown in Table 4. The intestinal weight increased significantly (*P <  0.01*) in the birds fed a diet supplemented with 400 mg/kg diet of vitamin C alone or in combination with a 10 g safflower oil/kg diet. The weight of the thymus and the bursa of Fabricius significantly increased (*P <  0.05*) after the dietary inclusion of vitamin C alone. The liver weight increased significantly (*P <  0.01*) in the group of birds fed a diet containing 5 g safflower oil/kg. Dietary inclusion of safflower oil and vitamin C had no significant effect on the gizzard, spleen, heart, and carcass weights (*P > 0.05*).

**Table 4:** The effect of inclusion of safflower oil and vitamin C in the broiler chicken diet on the carcass traits % relative to the live body weight

Item	Vitamin C level (mg/kg diet)		Safflower oil level (gm/kg diet)			Vitamin C × Safflower oil						SEM	*P-value*		
	0	400	0	5	10	0Vit.C+ 0Saff. Oil	0Vit.C + 5 Saff. Oil	0Vit.C+ 10 Saff. Oil	400Vit.C+ 0Saff. Oil	400Vit.C+ 5 Saff. Oil	400Vit.C+ 10Saff. Oil		Vit. C	Saff. oil	Vit. C x Saff. oil
Intestine	5.95	6.40	5.85	6.16	6.53	5.13^b^	6.43^ab^	6.28^ab^	6.55^a^	5.87^ab^	6.77^a^	0.16	0.17	0.32	0.01
Spleen	0.10	0.11	0.09	.011	0.11	0.09	0.09	0.10	0.08	0.13	0.11	0.008	0.50	0.41	0.67
Bursa	0.11^a^	0.16^a^	0.17^a^	0.12^ab^	0.11^b^	0.15^ab^	0.08^b^	0.08^b^	0.18^a^	0.15^ab^	0.13^ab^	0.01	< 0.01	0.04	< 0.01
Thymus	0.43^b^	0.57^a^	0.4	0.50	0.51	0.33^b^	0.43^ab^	0.51^ab^	0.62^a^	0.56^ab^	0.51^ab^	0.02	0.01	0.90	0.03
Gizzard	2.72	2.43	2.46	2.59	2.66	2.48	2.93	2.73	2.44	2.25	2.58	0.07	0.05	0.58	0.09
Liver	2.20	2.09	2.08^b^	2.44^a^	1.90^b^	2.03^bc^	2.56^a^	1.99^bc^	2.13^abc^	2.32^ab^	1.80^c^	0.06	0.44	< 0.01	< 0.01
Carcass	63.60	63.15	63.93	62.75	63.45	64.81	61.93	64.05	63.04	63.56	62.85	0.41	0.60	0.53	0.47
Heart	0.43	0.45	0.46	0.42	0.45	0.42	0.42	0.45	0.49	0.41	0.45	0.01	0.66	0.60	0.81

^a, b, c^ Means within the same row carrying different superscripts are significantly different at (*P < 0.05*)

### Behavioral observations

The effect of dietary supplementation with safflower oil and vitamin C on the broilers’ behavior is represented in Table 5. The results showed that the feeding, resting, and comfort behavior significantly increased (*P < 0.05*) in the birds fed a diet supplemented with vitamin C than in the birds fed a non-supplemented diet. The birds fed on a diet supplemented with vitamin C engaged in significantly less feather pecking (*P = 0.02*). Dietary inclusion of Safflower oil had a positive effect on most of the observed behaviors. The feather preening (comfort behavior) was significantly increased in the group fed a diet enriched with vitamin C and safflower oil (*P = 0.04*).

**Table 5:** The effect of inclusion of safflower oil and vitamin C in the broiler chicken diet on the behavioral activities

	Vitamin C level (mg/kg diet)		Safflower oil level (g/kg)			Vitamin C × Safflower oil						SEM	*P-value*		
	0	400	0	5	10	0Vit.C+ 0 Saff. Oil	0Vit.C + 5 Saff. Oil	0Vit.C+ 10 Saff. Oil	400Vit.C+ 0 Saff. Oil	400Vit.C+ 5 Saff. Oil	400Vit.C+ 10 Saff. Oil		Vit. C	Saff. oil	Vit. C x Saff. oil
Feeding	48.99^b^	79.86^a^	48.76^b^	62.56^ab^	81.93^a^	32.27	49.96	64.76	65.28	75.15	99.15	9.6	0.001	0.015	0.89
Drinking	33.85	37.82	28.44^b^	24.35^b^	54.71^a^	32.22	16.65	52.68	24.67	32.06	56.73	8.51	0.6	0.003	0.49
Resting	129.6^b^	211.04^a^	115.94^b^	172.9^b^	222.12^a^	70.28	148.5	170.21	161.6	197.48	274.03	12.55	< 0.01	< 0.01	0.41
Standing	53.65	44.4	68.69^a^	51.26^ab^	27.12^b^	81.88	42.1	36.98	55.51	60.43	17.27	10.6	0.33	0.003	0.12
Walking	46.44	47.27	50.08	54.74	35.76	32.33^b^	62.61^a^	44.40^b^	67.83 ^a^	46.86^b^	27.13^b^	9.2	0.9	0.15	0.02
Feather preening	27.99^b^	57.97^a^	30.89^b^	45.63^ab^	52.42^a^	24.78^b^	33.3^b^	25.9^b^	37.0^b^	57.96^ab^	78.94^a^	7.2	< 0.01	0.03	0.04
Others comfort	15.77^b^	34.94^a^	23.35	28.59	27.13	15.87	16.65	14.8	30.83	34.53	39.46	5.9	< 0.01	0.85	0.75
Foraging	25.56	26.72	35.95^a^	30.76^a^	11.7^b^	29.98	43.02	3.68	41.93	18.5	19.73	3.58	0.86	0.01	0.02
Feather pecking	5.45	1.64	5.89	2.75	1.99	8.09	5.5	2.76	3.68	0.62	1.23	0.65	0.02	0.147	0.59

^a, b^ Means within the same row carrying different superscripts are significantly different (*P* < 0.05)

### Fatty acid composition of the breast muscle

Table 6 highlights the effect of dietary inclusion of safflower oil and vitamin C on the breast muscle’s fatty acid composition. In general, safflower oil inclusion resulted in a significant increase (*P < 0.05*) in the levels of stearic acid, linoleic acid, saturated fatty acids, and omega-3 fatty acids. Meanwhile, the omega-3/omega-6 fatty acids ratio “ω-3/ω-6 ratio” increased (*P = 0.01*) only in the group fed a diet containing 10 g of safflower oil/kg diet. The inclusion of vitamin C had no significant effect (*P > 0.05*) on the fatty acid composition of the breast muscle.

**Table 6:** The effect of inclusion of safflower oil and vitamin C in the broiler chicken diet on the fatty acid composition of the breast muscle (% of total fatty acids)

Item	Vitamin C level (mg/kg diet)		Safflower oil level (gm/kg diet)			Vitamin C × Safflower oil						SEM	*P-value*		
	0	400	0	5	10	0Vit.C+ 0Saff. Oil	0Vit.C + 5 Saff. Oil	0Vit.C+ 10 Saff. Oil	400Vit.C+ 0Saff. Oil	400Vit.C+ 5 Saff. Oil	400Vit.C+ 10Saff. Oil		Vit. C	Saff. oil	Vit. C x Saff. oil
C14:0	0.13	0.14	0.13	0.14	0.15	0.12	0.14	0.14	0.14	0.15	0.15	0.01	0.40	0.40	0.79
C16:0	2.60	2.64	2.51	2.66	2.69	2.48	2.65	2.68	2.54	2.66	2.70	0.20	0.84	0.64	0.97
C16:1	1.07	1.08	1.05	1.09	1.09	1.04	1.09	1.08	1.06	1.09	1.10	0.04	0.74	0.45	0.89
C18:0	1.17	1.21	1.11^b^	1.22^a^	1.23^a^	1.08	1.21	1.21	1.15	1.22	1.25	0.04	0.29	0.01	0.07
C18:1	5.06	5.17	5.09	5.11	5.14	5.00	5.07	5.09	5.18	5.15	5.18	0.25	0.49	0.97	0.99
C18:2	2.02	2.06	1.96^b^	2.07^a^	2.08^a^	1.93	2.06	2.06	2.00	2.07	2.10	0.04	0.29	0.01	0.07
C18:3	0.16	0.24	0.12	0.23	0.24	0.06	0.20	0.21	0.18	0.26	0.27	0.04	0.07	0.06	0.08
C20:4	0.08	0.10	0.08	0.10	0.11	0.06	0.09	0.10	0.09	0.10	0.11	0.02	0.36	0.52	0.82
SFA	4.13	4.18	4.08^b^	4.18^a^	4.20^a^	4.04	4.17	4.18	4.12	4.20	4.21	0.04	0.23	0.01	0.08
MUFA	6.23	6.39	6.18	6.35	6.41	6.10	6.27	6.32	6.26	6.42	6.49	0.25	0.46	0.66	0.94
PUFA	2.16	2.25	0.41	0.43	0.50	2.05	2.19	2.25	2.16	2.28	2.31	0.23	0.67	0.78	0.98
ω-3	0.13	0.19	0.09^b^	0.18^a^	0.21^a^	0.04^b^	0.15^ab^	0.18^a^	0.13^ab^	0.20^a^	0.24^a^	0.02a	0.06	0.01	0.006
ω-6	2.04	2.06	2.02	2.06	2.07	2.01	2.03	2.07	2.03	2.08	2.07	0.22	0.91	0.97	1.00
ω-3/ ω-6	0.07	0.10	0.04^b^	0.09^ab^	0.11^a^	0.02	0.09	0.10	0.07	0.10	0.12	0.01	0.22	0.01	0.07
S/U	0.49	0.49	0.50	0.49	0.49	0.50	0.50	0.49	0.49	0.49	0.48	0.02	0.69	0.91	0.99

^a, b^ Means within the same row carrying different superscripts are significantly different at (*P* < 0.05)

Myristic acid(C14:0), Palmitic acid (C16:0), Palmitoleic acid (C16:1), Stearic acid (C18:0), Vaccenic acid(C18:1), Linoleic acid (C18:2), α-Linolenic acid (C18:3), Arachidonic acid (C20:4), *SFA* saturated fatty acids, *MUFA* monounsaturated fatty acids, *PUFA* polyunsaturated fatty acids, ω-3:Omega− *3* fatty acids, ω-6:Omega− *6* fatty acids, *S/U* saturated/unsaturated fatty acids, *MDA* malondialdehyde

### Blood biochemical parameters

The effect of dietary inclusion of safflower oil and vitamin C on the antioxidant defense system, selective immunological parameters, and liver and kidney function tests is shown in Table 7. In the current study, the birds’ antioxidant status was assessed by measuring the serum levels of CAT and SOD and the breast muscle concentration of GSH and MDA. The results revealed a significant increase (*P < 0.05*) in the serum levels of CAT, SOD, and GSH in the vitamin C-supplemented diets. Safflower oil inclusion had no significant effect (*P > 0.05*) on these previously mentioned parameters. The interaction between vitamin C and safflower oil did not cause any significant effect (*P > 0.05*) on these parameters. The muscle MDA level was decreased significantly (*P < 0.01*) by safflower oil inclusion. Interaction between vitamin C and safflower oil resulted in a significant decrease (*P < 0.01*) in the MDA level in the muscles of birds fed diets supplemented with safflower oil and vitamin C.

**Table 7:** The effect of inclusion of safflower oil and vitamin C in the broiler chicken diet on the antioxidant defense system, selective immunological parameters and liver, and kidney function tests

Item	Vitamin C level (mg/kg diet)		Safflower oil level (gm/kg diet)			Vitamin C × Safflower oil						SEM	*P-value*		
	0	400	0	5	10	0Vit.C+ 0Saff. Oil	0Vit.C + 5 Saff. Oil	0Vit.C+ 10 Saff. Oil	400Vit.C+ 0Saff. Oil	400Vit.C+ 5 Saff. Oil	400Vit.C+ 10Saff. Oil		Vit. C	Saff. oil	Vit. C x Saff. oil
Antioxidant status															
Serum CAT (U/L)	567.44^b^	580.00^a^	571.16	573.50	576.50	563.33	567.00	572.00	581.00	580.00	579.00	3.17	0.04	0.81	0.52
Serum SOD (U/ml)	25.94^b^	32.85^a^	26.60	29.81	31.78	23.40	25.06	29.36	29.80	34.56	34.20	1.49	0.01	0.38	0.14
Serum GSH (mmol/L)	4.14^b^	6.57^a^	4.50	5.36	6.21	3.40	3.96	5.06	5.60	6.76	7.36	0.55	0.02	0.47	0.25
Muscle MDA (μmol/gm)	4.61	3.60	5.34^a^	3.70^b^	3.27^b^	6.35^a^	4.01^b^	3.47^b^	4.32^b^	3.40^b^	3.07^b^	1.81	0.08	< 0.01	< 0.01
Immunological status															
IgA (ng/ml)	55.80^b^	66.58^a^	58.48	60.71	64.38	53.53^b^	56.73^b^	57.13^ab^	63.43^ab^	64.70^ab^	71.63^a^	2.14	0.001	0.43	0.01
Complement-3 (ng/ml)	134.66^b^	147.88^a^	137.50	140.83	145.50	131.00^b^	135.00^a^	138.00^ab^	144.00^ab^	146.66^ab^	153.00^a^	5.23	0.00	0.32	< 0.01
Cortisone (ng/ml)	9.95	10.07	10.03	10.01	10.00	9.90	10.03	9.93	10.06	10.00	10.16	0.07	0.44	0.98	0.95
Liver function															
ALT (U/L)	12.44	11.77	12.00	11.83	12.50	12.33	12.00	13.00	11.66	11.66	12.00	0.71	0.65	0.93	0.99
AST (U/L)	34.33	34.33	35.00	33.83	34.16	35.00	34.33	33.66	35.00	33.33	34.66	0.82	1.00	0.85	0.99
Kidney function															
Uric acid (mg/dl)	4.92	5.01	5.41	5.06	4.41	5.23	4.50	5.03	5.60	5.63	3.80	0.36	0.90	0.54	0.73
Creatinine (mg/dl)	0.69	0.71	0.71	0.70	0.69	0.71	0.69	0.67	0.72	0.72	0.71	0.01	0.3	0.71	0.88

^a, b^ Means within the same row carrying different superscripts are significantly different at (*P < 0.05*)

The immunological parameters represented by the serum levels of IgA and complement C3 were also assessed. The dietary inclusion of vitamin C resulted in significantly higher levels (*P < 0.01*) of these two components. The interaction between vitamin C and safflower oil resulted in a significant increase (*P < 0.05*) in the serum levels of IgA and complement C3 in the birds fed a diet supplemented with 400vit.C + 10 Saff. Oil (400 mg vitamin C/kg diet+ 10 g safflower Oil/kg diet). The serum levels of cortisone, ALT, AST, urea, and creatinine were not significantly (*P* > 0.05) affected by the dietary inclusion of safflower oil and vitamin C.

## Discussion

This study assessed the effect of safflower oil and vitamin C inclusion in the broiler chicken diets on the growth performance, behavior, amino acids AID%, carcass traits, the fatty acid composition of breast muscle, intestinal histology, immunological and antioxidant status of broiler chickens. The results revealed a positive effect of dietary inclusion of safflower oil and vitamin C on the BW, BWG, FI, and RGR of the broiler chickens. This may be attributed to the effect of their isolated or combined action on increasing the AID% of amino acids, improving the intestinal health, and increasing its absorptive surface, as demonstrated in our study. Dietary supplementation with safflower oil and vitamin C improved the intestinal histology and increased the villous height and width, crypt depth, villous height/crypt depth ratio, mucosal thickness, goblet cell count, and intra-epithelium lymphocytic lick cells infiltrations. Absorptive efficiency is controlled by the condition of the intestinal absorptive surface. Morphologically, the crypt depth and intestinal villi length indicate the intestinal absorptive capacity [34]. Increased intestinal villi length leads to an increase in the mucosal surface area, and consequently, increased nutrient absorption [35–38]. The improved intestinal morphology observed in the current study may be attributed to the PUFA composition of safflower oil [39]. Moreover, ascorbic acid can affect intestinal health by enhancing tensile strength, epithelial formation, and internal protein flow [40, 41].

Several studies on rats and broilers investigated the effect of FA composition on fat digestibility. Better utilization and decreased fecal energy losses were reported from unsaturated fats compared to those from saturated fats. This resulted in an increased ME for the unsaturated fats [42–45] and additional energy utilization [46]. An expected higher fat deposition was observed due to the increased triglyceride storage in the adipose tissue. Shimomura, et al. [47] reported a decrease in the accumulated body fat in rats fed a diet supplemented with safflower oil compared to those fed a diet supplemented with beef tallow. Korver, et al. [48] observed an increase in the BWG in chicks fed diets containing safflower oil, tallow, and corn oil compared to those fed diets with cellulose and fish oil. Several studies reported an improved efficiency in broiler chickens’ performance by the dietary supplementation of vitamin C [49–52]. Gouda, et al. [31] reported improvement in the growth performance, insulin growth factor 1, and thyroid hormone levels of the broilers by supplementing the diet with vitamin C (200 mg/kg) alone or along with folic acid (1.5 mg/kg). Sahin and Kucuk [53] stated that supplementation with 200 mg/kg diet of vitamin C, and not 100 mg/kg diet, resulted in increased FI, BW, and feed efficiency of Japanese quails. They also reported increased bird performance, nutrient digestibility, and carcass traits by dietary supplementation of vitamin C and/or vitamin E. Ascorbic acid improved the feed efficiency by increasing the nutrient digestibility and controlling the deficiency of minerals and vitamins [54]. Dietary supplementation with vitamin C increases the iron absorption by reducing Fe^3+^ to Fe^2+^, which is more absorbed by the intestine; thus, vitamin C increases the resistance to infections. Protein modifications caused by oxidative lesions may inhibit the pancreatic enzymes and dietary protein resistance to digestion. Hence, dietary supplementation with antioxidants such as vitamin C could prevent oxidative protein denaturation, and consequently, improve nutrient digestibility and feed efficiency [41, 55, 56]. Ciftci, et al. [57] demonstrated that ascorbate supplementation resulted in insignificant effects on birds’ growth and egg production. They attributed this to the insufficiency of the vitamin C dosage required to overcome heat stress. Moreover, improved FCR was reported in broilers subjected to heat stress after feeding them a vitamin C-supplemented diet [58, 59]. Attia, et al. [27] reported improvements in the BWG and FCR of heat-stressed broiler chickens by dietary supplementation of vitamin C alone and combination with vitamin E and probiotics.

Behavior is an important indicator for evaluating the well-being of animals. Our results showed the benefits of dietary supplementation of vitamin C and safflower oil on the broiler chickens’ behavioral observation and comfort level. Vitamin C supplementation improved their feeding behavior. Safflower oil inclusion improves the ingestive behavior (feeding and drinking). Chand, et al. [60] reported an increase in the feed intake of birds fed a diet enriched in ascorbic acid and Zn. El Iraqi, [61] stated that the patterns depicting comfort behavior increased by enrichment of the diet with anti-stress additive/s. Feather pecking in birds is considered a harmful activity. Our results revealed that enrichment of the diet with vitamin C positively reduces this unfavorable behavior. The study of Nosrati, et al. [62] assessed the effect of adding vitamin C, probiotics, antibiotics, herbal extract, and organic acid into the drinking water of broiler chickens and reported improved body weight of birds by supplementing the drinking water with vitamin C alone or with the antibiotic.

There is a great focus on researching foods containing polyunsaturated fatty acids at higher levels because of their benefits to human health [3, 6–8]. Raza, et al. [63] reported that supplementing the Hy-Line White Leghorn diet with a mixture of 25% hempseed and 2% turmeric and ginger enriched the egg yolk with PUFA, ω-3, and ω-6. The current study results indicated that safflower oil inclusion improved the fatty acid composition of the diet and increased the PUFA content of the breast muscle in the carcass. Birds cannot synthesize linoleic and linolenic acids. Hence, their occurrence and quantity in the body lipids depend on their supplementation through the diet and on the rate of tissue oxidation [64]. Kirchgessner, et al. [65] reported an increase in breast muscle fat content by increasing the dietary linoleic acid levels. Plant-rich oils contain high levels of polyunsaturated fatty acids (PUFA) but vary in essential FA composition. Kishawy, et al. [3] reported an increase in the alpha-linolenic acid content in the breast muscle of broiler chickens after replacing soybean oil with linseed oil in the diets. PUFA is incorporated in the phospholipids [66] of the muscle fat rather than adipose tissue fat [67]. PUFA feeding probably leads to increased dietary fatty acid oxidation as they increase the expression of acyl-CoA oxidase, the chief enzyme of β-oxidation, in the peroxisomes [68, 69]. Further, PUFA oxidation results in internal fatty acid synthesis from carbohydrates; this incurs a greater energy expenditure than the direct deposition of fatty acids from the diet [70]. Hence, the dietary addition of antioxidants is required to prevent the β-oxidation of these FA.

One of the most important findings from our study was that the mixture of safflower oil and vitamin C combined both these supplements’ features. The final product was an increased bodyweight that was enriched by essential fatty acids and antioxidant substances. A significant increase was observed in the serum levels of CAT, SOD, and GSH in chickens fed with vitamin C-supplemented diets in the current study. A significant decrease was observed in the MDA level in the muscles of birds fed diets supplemented with safflower oil and/or vitamin C. Ascorbate is involved in the regeneration of reduced glutathione from the oxidative form in the cytoplasm; it also aids in the regeneration of tocopherol through a non-enzymatic reaction [71]. Gouda, et al. [31] demonstrated an improved total antioxidant capacity, catalase activity, and SOD activity of broiler chickens under heat stress when they were fed a diet supplemented with vitamin C (200 mg/kg) alone or in combination with folic acid (1.5 mg/kg).

Similarly, dietary supplementation with Vitamin C reduced the serum level of MDA, as observed by Sahin, et al. [72]. Öztürk-Ürek, et al. [73] observed increased levels of SOD and GPx in adult chickens fed ascorbic acid-supplemented diets. Florou-Paneri, et al. [74] observed significantly lower MDA levels in the chilled tissue of raw breast muscles of chickens fed a vitamin C-supplemented diet.

The chicken immune response showed an improvement after dietary supplementation with vitamin C and with the combination of 400vit.C + 10 Saff. Oil; this is concluded from the higher serum levels of IgA and complement C3. Gouda, et al. [31] demonstrated an increased antibody titer against the Newcastle disease virus and decreased heterophil/lymphocytes in broiler chickens by supplemental vitamin C (200 mg/kg) alone or in combination with folic acid (1.5 mg/kg). Chand, et al. [60] observed that broiler chickens fed a diet supplemented with ascorbic acid, and Zn showed increased weights of the spleen, thymus, and bursa. They also reported an improvement in the cellular and humoral immunity in birds fed a diet supplemented with 300 mg/kg of ascorbic acid. Bendich [75] confirmed that ascorbic acid is necessary for the systemic availability of immunoglobulins and interferons. Additionally, ascorbic acid supplementation increased the levels of CD^8+^ and IgM [76]. Asli, et al. [77] reported an insignificant increase in the immune response by dietary supplementation of vitamin C and vitamin E.

The enhanced immune status with vitamin C supplementation can be attributed to its role in reducing the adrenocorticotropic hormone that has a suppressive and cytotoxic effect on the immune function [78]. Moreover, ascorbic acid increases the differentiation of the lymphoid organ by improving the activity of the hexose monophosphate pathway, which increases the production of antibodies [79]. Furthermore, the improved immune status can be attributed to the crucial role of PUFA composition in safflower oil that has been reported to have immune health-enhancing properties in poultry [80]. Dietary fat impacts the inflammatory response by modulating the production of cytokines [81]. Feeding with n-6 fatty acids increased the cell-mediated immune response, while n-3 fatty acids increased the antibody-mediated immune response [82].

Dietary supplementation with safflower oil and vitamin C did not affect the serum levels of cortisone, ALT, AST, urea, and creatinine. Similar results were reported in [83]. Similarly, Attia, et al. [27] reported no significant effect of vitamin supplementation on the serum levels of AST and ALT.

## Conclusions

The current results indicated that a dietary combination of safflower oil and vitamin C improved the growth performance by improving the AID% of amino acids and intestinal histology. This combination improved the immune status, antioxidant status, the ingestive, resting, and feather preening behavior of broiler chickens; the best supplementation level was 400vit.C + 10 Saff. Oil. Safflower oil inclusion increased stearic acid levels, linoleic acid, saturated fatty acids, and omega-3 fatty acids in the breast muscle. This also leads to increased customer acceptance.

## Methods

### Birds

Three hundred 1-day-old chicks (Ross 308 broiler) were procured from a commercial chick producer (Dakahlia Poultry, Mansoura, Egypt) for the experiments. Before starting the investigation, they were subjected to a 3-day adaptation period. During this period, they were fed the control diet, and their body weight reached an average of 58.25 ± 0.19 g. The study was conducted at the Faculty of Veterinary Medicine, Zagazig University, Egypt. The Ethics approved the experimental protocol of the Institutional Animal Care and Use Committee of Zagazig University, Egypt (ZUIACUC–2019). All animal experiments were performed following the recommendations described in “The Guide for the Care and Use of Laboratory Animals in scientific investigations”. Chicks were housed within the same sanitary, environmental, and managerial conditions throughout the experimental period. The chicks were reared at 10 bird/m^2^ stocking density. The light regimen in all experimental pens was maintained at 23 L: 1 D h for the first 3 days, followed by 20 L: 4 D h until the end of the experiment. The initial ambient temperature was about 32 °C during the first week and then gradually reduced every week by 2 °C until it reached 22 °C; the relative humidity (RH%) range was 65–75%. Birds were reared in a naturally ventilated open house with sawdust as litter. General health and vaccination practices were followed against the New Castle (on 4th and 14th day) and Gumboro diseases (on 7th and 22nd day). The health condition of all chicks was closely monitored. A daily check was performed on the chicks for any health challenges. After completion of the study, all remaining chickens were released.

### Experimental design and diets

Vitamin C (ROVIMIX® STAY-C®35) was primarily produced for use in the feed as a stable source of vitamin C. Safflower oil was extracted from safflower seeds by a screw press expeller according to the method described in [84].

Birds were randomly assigned to a 2 × 3 factorial design consisting of six experimental groups with five replicates (10 chicks/replicate). The experimental groups consisted of two vitamin C levels (0 and 400 mg/kg diet) and three levels of safflower oil (0, 5, and 10 g/kg diet). The experiment lasted for 35 days and was allocated three periods: starter (from 4- ten days), grower (from 11 to 23 days), and finisher period (from 24 to 35 days). Freshwater and feed were offered for ad libitum consumption throughout the experiment. The proximate chemical composition of the experimental diets is shown in Table 8. Iso-energetic and iso-nitrogenous diets were formulated following Ross 308 broiler nutrition specifications, Aviagen [85].

**Table 8:** Proximate and chemical composition of the experimental diets (g/kg)

Ingredients	Starter period (4–10 d)						Grower period (11–23 d)						Finisher period (24–35 d)					
	T1	T2	T3	T4	T5	T6	T1	T2	T3	T4	T5	T6	T1	T2	T3	T4	T5	T6
yellow corn	556.4	556.4	556.4	556.4	556.4	556.4	594.3	594.3	594.3	594.3	594.3	594.3	624.3	624.3	624.3	624.3	624.3	624.3
Soybean meal, 48%	320	320	320	320	320	320	280	280	280	280	280	280	231.3	231.3	231.3	231.3	231.3	231.3
Corn gluten, 60%	59.8	59.8	59.8	59.4	59.4	59.4	55.7	55.7	55.7	55.3	55.3	55.3	65.7	65.7	65.7	65.3	65.3	65.3
Safflower oil	0	5	10	0	5	10	0	5	10	0	5	10	0	5	10	0	5	10
Soybean oil	20	15	10	20	15	10	30	25	20	30	25	20	40	35	30	40	35	30
Vitamin C	0	0	0	0.4	0.4	0.4	0	0	0	0.4	0.4	0.4	0	0	0	0. 4	0. 4	0. 4
Calcium carbonate	13	13	13	13	13	13	12	12	12	12	12	12	10.5	10.5	10.5	10.5	10.5	10.5
Calcium dibasic phosphate	15	15	15	15	15	15	13	13	13	13	13	13	13	13	13	13	13	13
Common salt	1.5	1.5	1.5	1.5	1.5	1.5	1.5	1.5	1.5	1.5	1.5	1.5	1.5	1.5	1.5	1.5	1.5	1.5
Premix^*^	3	3	3	3	3	3	3	3	3	3	3	3	3	3	3	3	3	3
DL-Methionine, 98%	2.3	2.3	2.3	2.3	2.3	2.3	2	2	2	2	2	2	1.8	1.8	1.8	1.8	1.8	1.8
Lysine, Hcl, 78%	4.7	4.7	4.7	4.7	4.7	4.7	4.2	4.2	4.2	4.2	4.2	4.2	4.6	4.6	4.6	4.6	4.6	4.6
Choline	0.7	0.7	0.7	0.7	0.7	0.7	0.7	0.7	0.7	0.7	0.7	0.7	0.7	0.7	0.7	0.7	0.7	0.7
Threonine	1	1	1	1	1	1	1	1	1	1	1	1	1	1	1	1	1	1
Phytase	0.1	0.1	0.1	0.1	0.1	0.1	0.1	0.1	0.1	0.1	0.1	0.1	0.1	0.1	0.1	0.1	0.1	0.1
NaCo3	2.5	2.5	2.5	2.5	2.5	2.5	2.5	2.5	2.5	2.5	2.5	2.5	2.5	2.5	2.5	2.5	2.5	2.5
Chemical composition (g/kg)**																		
ME kcal/kg diet	3012.65	3012.65	3012.65	3011.16	3011.16	3011.16	3108.199	3108.19	3108.19	3106.71	3106.71	3106.71	3213.92	3213.92	3213.92	3212.43	3212.43	3212.43
Crude Protein%	23.48	23.48	23.48	23.46	23.46	23.46	21.57	21.57	21.57	21.54	21.54	21.54	20.14	20.14	20.14	20.11	20.11	20.11
Calcium	9.7	9.7	9.7	9.7	9.7	9.7	8.7	8.7	8.7	8.7	8.7	8.7	8.1	8.1	8.1	8.1	8.1	8.1
Available P	4.8	4.8	4.8	4.8	4.8	4.8	4.3	4.3	4.3	4.3	4.3	4.3	4.1	4.1	4.1	4.1	4.1	4.1
Lysine	14.4	14.4	14.4	14.4	14.4	14.4	12.9	12.9	12.9	12.9	12.9	12.9	11.9	11.9	11.9	11.9	11.9	11.9
Methionine	5.6	5.6	5.6	5.6	5.6	5.6	5.1	5.1	5.1	5.1	5.1	5.1	4.8	4.8	4.8	4.8	4.8	4.8
Threonine	9.7	9.7	9.7	9.7	9.7	9.7	8.8	8.8	8.8	8.8	8.8	8.8	8.1	8.1	8.1	8.1	8.1	8.1

T1: basal diet without any additives (control group), T2:basal diet+ 5 g/kg diet safflower oil, T3: basal diet+ 10 g/kg diet safflower oil, T4: basal diet+ vitamin C (400 mg/kg diet), T5:basal diet+ vitamin C (400 mg/kg diet) + 5 g/kg diet safflower oil, T6: basal diet+ vitamin C (400 mg/kg diet) + 10 g/kg diet safflower oil

* Premix per kg of diet: vitamin A, 1500 IU; vitamin D3, 200 IU; vitamin E, 10 mg; vitamin K3, 0.5 mg; thiamine, 1.8 mg; riboflavin, 3.6 mg; pantothenicacid, 10 mg; folicacid, 0.55 mg; pyridoxine, 3.5 mg; niacin, 35 mg; cobalamin, 0.01 mg; biotin, 0.15 mg; Fe, 80 mg; Cu, 8 mg; Mn, 60 mg; Zn, 40 mg; I, 0.35 mg; Se, 0.15 mg

^**^ According to Ross manual Guide, Aviagen [85]

### Growth performance

The initial weight of the birds was obtained on the 4th day of age; the bodyweight “BW” was then recorded at the end of the starter, grower, and finisher stages (10, 23, and 35 days, respectively) to estimate the average BW of the birds in each group. The body weight gain “BWG” was calculated as W2–W1, where W2 is the final BW in the intended period, and W1 is the initial BW in the same period. Feed intake “FI” of each replicate was recorded as the difference between the weight of the feed offered and the residues left and then divided by the number of birds in each replicate to find out the average feed intake per bird. According to an earlier study, feed conversion ratio “FCR” was estimated at the end of each stage [86]. The relative growth rate (RGR) was calculated at the end of the experiment by the equation described in [87].

### Apparent ileal digestibility coefficient “AID%” of amino acids

#### Diet and birds

To determine the amino acid ileal digestibility, titanium dioxide was added to the feed at 0.5% dosage (5 kg/t of feed) for 5 days. It is an indigestible marker substance, characterized by a high recovery rate of almost 100%, and does not affect the nutrients’ digestibility. Each experimental diet was offered ad libitum to three replicates (five birds/replicate) of broiler chickens from 35 to 40 days of age. At the end of the digestibility trial, the birds were euthanized using cervical dislocation according to [88], and the contents of the lower half of the ileum were collected into plastic containers, pooled, frozen directly after collection, and then freeze-dried. Dried samples were ground and stored in airtight containers at −20 °C for chemical analysis.

#### Analysis of amino acid concentration

The concentration of amino acids in the diet and the ileal digesta samples were estimated by the method described in [89, 90]. Tryptophan was determined separately using the method of [91]. Titanium dioxide was estimated using the procedures of [92]. Amino acid AID% was estimated by the following equation: AID (%) = 100 - [(Ti_(diet)_ × AA_(ileum))_/(TI_(ileum)_ × AA_(diet)_) × 100].

where Ti (diet) is the titanium dioxide concentration in the diet, Ti (ileal) is the titanium dioxide concentration in the ileal digesta, AA (ileal) is the concentration of the test AA in the ileal digesta, and AA (diet) is the concentration of the test AA in the diet.

#### Histological examination of the gut

The intestinal specimens of the broilers were collected and immediately fixed in 10% buffered neutral formalin solution for 48 h, dehydrated in a gradual ascending concentration of ethanol (70, 80, 90, 95, and 100%), cleared in xylene, and embedded in paraffin. Five-micron thick paraffin was sliced using a microtome (Leica RM 2155, England). The sections were prepared and then routinely stained with hematoxylin and eosin and examined microscopically [93]. Morphometric analysis was performed by the camera microscope software AmScope® (AmScope digital camera-attached Ceti England microscope) as follows: Villus height measured (μm) from the tip to the base of the villus. The diameter, muscular thickness, submucosa layer thickness, the number of goblet cells per area of the epithelium layer, and intraepithelial leucocytes were also calculated.

#### Carcass traits

At the end of the trial period of 35 days, five birds from each group were selected, maintained under overnight fasting condition, weighed, and euthanized using cervical dislocation, according to [88]. The feathers were pulled out, and the birds were disassembled and weighed to determine the proportion of dressing. The dressed carcass, liver, gizzard, intestines, heart, spleen, bursa, and abdominal fat were calculated as a percentage of the live BW. The relative weight of some organs was calculated according to [94].

#### Behavioral measurement

The broilers’ behavioral observation was recorded after fixing cameras over the experimental pens and recorded using a scan sampling technique [95]. Each group was observed twice daily (10 min each time), for six consecutive days per week (total 3 h/group/week) throughout the experimental period. The observation was carried out between 7 and 8 am and 2–3 pm.

The following behavioral parameters were observed and measured throughout the experiment: ingestive behavior (feeding, drinking), walking, standing, resting, comfort behavior (feather preening and other comforting activities that included wing flapping, head shaking, wing and leg stretches, and body shaking), foraging behavior, and feather pecking behavior as described in [96]. The number of birds performing each behavioral pattern was recorded in each scan (10 min), and the results were expressed as the percentage of birds performing the behavior/total number of observed birds [97].

#### Sample collection

At the end of the trial on day 35, birds were kept for fasting for 12 h, and blood samples were withdrawn from five birds. These birds were selected randomly from each experimental group and were euthanized using cervical dislocation, according to [88]. Blood samples were allowed to coagulate at room temperature or in the refrigerator for 1 h and then centrifuged at 3000 rpm for 15 min. The clear supernatant serum was transferred into dry, sterile, and labeled stopper vials and used for the clinical and biochemical tests, including liver and kidney function, antioxidant defense system, and selective immunological parameters. Samples from different parts of the gut were dissected for histological examination of the gut. Breast muscles were stored in a refrigerator at − 20 °C until the estimation of malondialdehyde (MDA) level and fatty acid composition was performed.

### Determination of the fatty acid composition of breast muscle

#### Extraction of lipids

Oils were extracted from the breast muscle using a solvent mixture of chloroform/methanol (2:1, v/v), which is suitable for the quantitative extraction of lipids [98].

#### Fatty acids separation and identification

Fatty acids in the extracted oil were determined, as mentioned for the safflower oil method [99].

#### Clinical and biochemical analyses

Serum activities of AST and ALT were estimated by the method of Reitman and Frankel [100], urea and creatinine were evaluated according to Coulombe and Favreau [101, 102], respectively, using a semi-automated Photometer (5010 V5+, RIELE GmbH & Co, Berlin, Germany) following the manufacturer’s protocol.

Chicken ELISA kits from MyBioSource (CAT. NO. MBS012469) and ABCAM (CAT. NO. AB157691) were used to determine the serum levels of cortisone and IgA, respectively. Meanwhile, the sandwich ELISA (enzyme-linked immunosorbent assay) kit from Life Span Biosciences, Inc. (CAT.NO.LS-F9287) was used for detecting the serum level of complement C3 following the instructions in the enclosed pamphlets.

Parameters of the antioxidant defense system, such as the serum level of CAT was estimated according to Aebi [103] and SOD, according to Nishikimi, et al. [104]. The serum level of reduced glutathione (GSH) was determined, according to an earlier study [105]. The Malondialdehyde (MDA) level was determined in the breast muscle according to Mcdonald and Hultin [106].

### Statistical analysis

Experimental data were analyzed using SPSS 18.0 for Windows (SPSS Inc., Chicago, IL, USA). The variation was assessed by two-way ANOVA. A factorial analysis was conducted with the factors in the model being the levels of vitamin C, safflower oil, and their interactions. Differences between the means were compared at a 5% probability using the post-hoc Tukey’s multiple comparison tests. Variation in the data was expressed as pooled SEM, and the significance level was set at *P* < 0.05.

## Data Availability

The datasets used and analyzed during the current study available from the corresponding author on reasonable request.

## Abbreviations

AID%: amino acid ileal digestibility coefficient
BW: body weight
BWG: body weight gain
FI: feed intake
FCR: feed conversion ratio
RGR: relative growth rate
PER: protein efficiency ratio
Ti: Titanium dioxide
AA: amino acid
HSP70: heat shock protein 70.
